# The burden of disease of fatal and non-fatal burn injuries for the full spectrum of care in the Netherlands

**DOI:** 10.1186/s13690-022-01020-z

**Published:** 2023-01-09

**Authors:** Inge Spronk, Margriet E. van Baar, Robert A. Verheij, Martien J. Panneman, Jan Dokter, Suzanne Polinder, Juanita A. Haagsma

**Affiliations:** 1 Department of Public Health, Erasmus MC, University Medical Centre Rotterdam, P.O. Box 2040, 3000 CA Rotterdam, the Netherlands; 2 Association of Dutch Burn Centres, Maasstad Hospital, Rotterdam, the Netherlands; 3 Nivel, Netherlands Institute for Health Services Research, Utrecht, The Netherlands; 4 Tranzo, Tilburg School of Social and Behavioural Sciences, Tilburg, The Netherlands; 5 Consumer Safety Institute, Amsterdam, The Netherlands; 6 Burn Centre, Maasstad Hospital, Rotterdam, the Netherlands

**Keywords:** Burn injuries, Burden of disease, Full spectrum of care

## Abstract

**Background:**

A comprehensive overview of the burden of disease of burns for the full spectrum of care is not available. Therefore, we estimated the burden of disease of burns for the full spectrum in the Netherlands in 2018, and explored whether the burden of disease changed over the past 5 years (2014-2018).

**Methods:**

Data were collected at four levels: general practice, emergency department, hospital, and mortality data. For each level, years lived with disability (YLD), years of life lost (YLL), and disability-adjusted life-years (DALY) were estimated using a tailored methodology.

**Results:**

Burns resulted in a total of 9278 DALYs in the Netherlands in 2018, comprising of 7385 YLDs (80%) and 1892 YLLs (20%). Burn patients who visited the general practice contributed most DALYs (64%), followed by deceased burn patients (20%), burn patients admitted to hospital (14%) and those treated at the emergency department (2%). The burden of disease was comparable in both sexes (4734 DALYs (51%) for females; 4544 DALYs (49%) for males), though the distribution of DALYs by level of care varied; females contributed more DALYs at the general practice level, and males at all other levels of care. Among children boys 0-4 years had the highest burden of disease (784 DALYs (9%)), and among adults, females 18-34 years old (1319 DALYs (14.2%)) had the highest burden of disease. Between 2014 and 2018 there was a marginal increase of 0.8% in the number of DALYs.

**Conclusions:**

Burns cause a substantial burden of disease, with burns requiring care at the general practice level contributing most DALYs. Information on burden of burns by the full level of care as well as by subgroup is important for the development of tailored burn prevention strategies, and the updated figures are recommended to be used for priority setting and resource allocation.

**Supplementary Information:**

The online version contains supplementary material available at 10.1186/s13690-022-01020-z.

## Background

Burn injuries are considered a leading cause of injury worldwide [1]. Due to substantial improvements in burn care, an increasing number of patients survive their injuries and live with the consequences [2]. Burns can have a considerable negative impact on physical and psychosocial functioning [3, 4].

Burn injuries also pose a burden on society. A well-established concept that assesses the impact of a health condition on population health is the burden of disease, which can be quantified by the disability adjusted life year (DALY) [5–7]. DALY is a combination of two figures: premature mortality, expressed in years of life lost (YLLs), and non-fatal health loss, expressed in years lived with disability (YLDs), which is adjusted for the severity of the disability by the use of disability weights [8–12].

The Global Burden of Disease (GBD) study provides estimates on the annual burden of disease of many conditions, including burns [13–15]. However, limitations of the GBD study for burn injuries include a sub-optimal linkage of data and disability weights [16], and the assumption that a greater proportion of patients with minor burns have lifelong consequences compared to patients with major burns, which is not plausible and contradicting to available evidence [17].

**Fig. 1:**
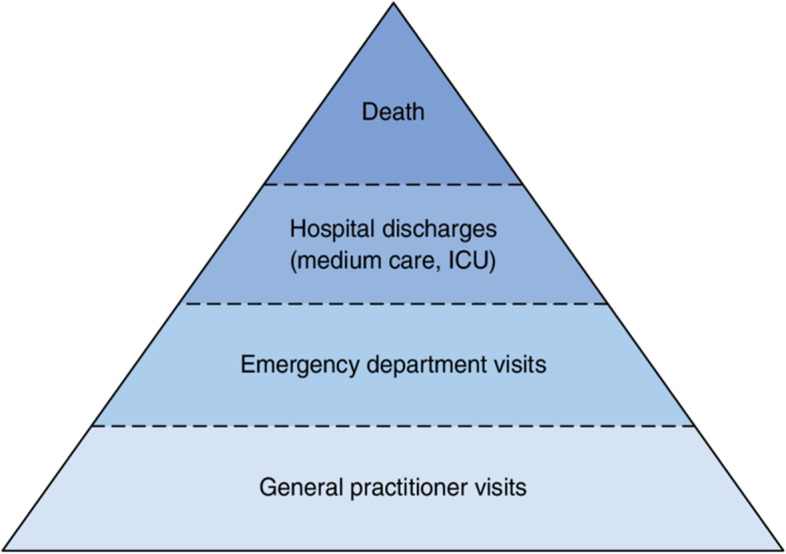
Injury surveillance pyramid from Polinder et al. [21] – copied with permission. ICU = Intensive Care Unit

An improved methodology to estimate the burden of disease of burns was recently developed: the INTEGRIS-burns method [18]. Important adaptations include tailored disability weights based on severity categorization of burn patients; a better substantiated proportion of patients with lifelong disability; and the use of a prolonged burn specific recovery period [18]. European and Western-Australian health-related quality of life (HRQL) data of 3401 patients were combined to derive disability weights for three burn injury groups based on percentage total body surface area (%TBSA) burned. Disability weights ranged from 0.046 (subgroup < 5% TBSA burned; > 24 months post-burn) to 0.497 (subgroup > 20% TBSA burned; 0–1 months post-burn). This method is fully explained elsewhere [18]. Up till now, the application of this refined method has been limited to the non-fatal burden of disease of patients treated in specialized burn care centres [18, 19]. These studies did not include the full spectrum of care and did not include mortality due to burns. Burn care is not limited to specialized burn centres. Care for less severe burn injuries is primarily provided by general practitioners (GPs) and general hospitals; i.e. hospitals without a specialized burn centre. In the Netherlands, the GP has a gatekeeper role for secondary care. An exception is an acute situation, where patients are immediately transferred to a hospital’s emergency department. For burn injuries, depending on the severity, this is either a general hospital or dedicated burn centre [20]. In the Netherlands, primary care is fully covered by the obligatory health care insurance, whereas for secondary care an annual deductible (385 euros in 2020) has to be paid; all other costs are covered by the health insurance. Due to this arrangement of health care, we expect no barrier for health care access for burn care. By including all levels of care, and burn-related mortality, an overview of the total burden of disease of burn injuries in the Netherlands can be estimated. The full spectrum of care can graphically be present by an injury surveillance pyramid (Fig. 1) [21, 22]. The primary aim of this study was to estimate the total burden of disease of fatal and non-fatal burn injury in the Netherlands for the full spectrum of care in 2018. The secondary aim was to explore whether the burden of disease of burns changed over the past 5 years (2014-2018).

## Methods

### Data sources

The following three sources were used to obtain the incidence data of burn injuries and treated: the Nivel Primary Care Database (Nivel-PCD) for GP visits, the Dutch Injury Surveillance System (DISS) for ED visits, the Dutch Hospital Discharge Registry (HDR) and the Dutch Burn Repository (DBR) R3 for hospital and burn centre admissions respectively. In addition, Cause-of-Death Statistics Netherlands was used to assess the mortality of burns in the Netherlands.

#### Ethical approval

According to Dutch law (https://english.ccmo.nl/investigators/legal-framework-for-medicalscientific-research/your-research-is-it-subject-to-the-wmo-or-not), ethics approval is not required for aggregated registry data as participants were not subject to procedures or required to follow rules of behaviour.

#### Incidence data

Data on general practice visits for burn injuries were obtained from Nivel-PCD (NZR00320.007). This database includes a national representative sample of 10% of the general practices (day care) and 65% of the general practice cooperatives (out-of-hours care). Patients with an ICPC-1 code S14 (burn injury) were selected (Table 1) [23]. Collected data were extrapolated to the whole Dutch population based on the size and distribution of the population of the general practices and general practice cooperatives based on a regularly applied method [24–27]. Because all Dutch citizens are registered at a general practice, Nivel-PCD also contains patients who do not visit their GP [28]. The age/sex specific number of patients included in Nivel-PCD was extrapolated to the number of persons in the specific age/sex categories in the Dutch population by using the Dutch population registry numbers from Statistics Netherlands [29]. This method has been used for the extrapolation of burn injuries in Dutch general practice before [24]. Fourteen percent of the patients visited both day care and out-of-hours care for the same burn injury [30], therefore the number of visits of out-of-hours care was lowered by 14%. Besides, 0.7% of all GP patients are generally referred to general hospital care, therefore the numbers were lowered by 0.7% [30]. In addition, age/sex specific rates of referrals from GP to burn centres per year were available and GP rates were lowered by these numbers to prevent double counting [31].

**Table 1:** Source and case definition for data on each level of the injury pyramid

Level of injury pyramid	Source	Case definition
Death	Cause-of-Death Statistics Netherlands	Deaths related to burns ICD-10 codes: W35-W40; X00-X19; X76-X77; X97-X98; Y26-Y27
Hospital admission	Dutch Hospital Discharge Registry	Hospital admission for a burn injury 2014: ICD-9 codes: 940-949 2015-2018: ICD-10 codes: T20-T32
	Dutch Burn Repository R3	Burn centre admission for a burn injury All patients in registry
ED visits	Dutch Injury Surveillance System	Patients with an ED visit but no hospital admission for a burn injury 2014: ICD-9 codes: 940-949 2015-2018: ICD-10 codes: T20-T32
GP visits	Nivel Primary Care Database	Patients with a GP visit for a burn injury ICPC-1 code: S14

Data on emergency department (ED) visits were obtained from DISS. DISS records patients who have been treated at the ED for an injury [32]. Data are collected from a national representative sample of EDs covering 12% of all Dutch ED visits [33, 34]. All burn-related ICD-9 codes (940-949) for 2014 and ICD-10 codes (T20-T32) for 2015-2018 were selected (Table 1) [35]. Data were extrapolated to the Dutch population by a standardized method [36, 37].

The Dutch Hospital Discharge Registry (HDR) includes all hospital admissions from all Dutch hospitals [38]. All burn-related ICD-9 codes (940-949) in 2014, and ICD-10 codes (T20-T32) in 2015-2018 were selected (Table 1). Patients who died during admission were not included to prevent double counting with mortality data.

Dedicated burn centres are included in the HDR, however, the Dutch Burn Repository (DBR-R3) includes more specific burn injury data than the HDR registry. Therefore, data of patients treated in a burn centre in 2014-2018 were derived from the DBR-R3 and removed from the HDR extraction (Table 1) [31]. Patients who died during admission were not included to prevent double counting with mortality data.

The collected data were summed into the predefined age- and sex-categories (i.e. 0-4 yr, 5-7 yr, 8-11 yr, 12-17 yr, 18-24 yr, 25-34 yr, 35-44 yr, 45-54 yr, 55-64 yr, 65-74 yr, ≥75 yr, separately for males and females) for each database separately. As patients can be treated at more than one healthcare setting, pathways of burn patients were studied to correct for double-counting as explained above. Patients were included at the highest level of care treated [30].

#### Mortality data

Age- and sex- specific mortality rates were derived from the Cause-of-Death Statistics Netherlands for 2014-2018 [39]. The Cause-of-Death statistics are based on official death certificates [40]. Causes of death related to burns were selected from this registry (ICD-10 codes W35-W40; X00-X19; X76-X77; X97-X98; Y26-Y27) (Table 1).

### Calculations

#### Years lived with disability

The calculation of years lived with disability (YLDs) is done for the short-term and long-term separately. Short-term (acute phase) is 12 months for patients without an admission, and 24 months for patients with a hospital/burn centre admission as patients with more severe burns have a prolonged recovery [4]. Consequently, long-term is > 24 months for patients with an admission, and > 12 months for those without admission. For the short-term, the calculation consisted of three steps: 1) calculating the incidence of burns in the predefined age- and sex-specific groups for each level of the injury surveillance pyramid. 2) for admitted patients: breaking down the incidence data into the homogenous burn categories based on percentage total body surface area (%TBSA) burned [18] (Additional file 1). In the HDR data we received, burn size was not available, therefore the distribution of %TBSA is adopted from the distribution in the burn centres [20]. 3) combining the grouped incidence data with the relevant disability weights and durations [18, 41]. Short-term YLDs were calculated by multiplying the disability weights with the corresponding duration of that specific disability weight, and with the incidence. For the long-term YLD calculations, the incidence data were combined with the proportion of patients with lifelong consequences, with the relevant disability weights, and with the age- and sex-specific remaining life expectancy (Additional file 1). Life expectancy at time of death was derived from the global burden of disease study 2017 [42, 43].

Short-term and long-term YLDs were combined to derive YLDs on group level. The group YLDs were summed to derive the overall YLDs of burn injuries. Both total YLDs as YLD per 1000 inhabitants were estimated. Country population data were derived from Statistics Netherlands [29]. Population numbers were extracted and used for each year (2014-2018) specifically.

#### Years of life lost

Age- and sex-specific life expectancy at age of death (in the predefined age/sex groups) was used to calculate the YLL [42, 43]. Mortality rates of the age- and sex-specific groups was multiplied by the specific life expectance of that group. Both total YLL as YLL per 1000 inhabitants were estimated.

#### Disability-adjusted life-years

The burden of disease of fatal and non-fatal burn injury in the Netherlands was expressed in DALYs for 2014-2018. For each year, the total YLDs were added up with the total YLLs, establishing the number of DALYs due to burn injuries. Total number of DALYs per year were estimated, as well as DALYs per 1000 inhabitants per year.

## Results

### Number of patients and deaths due to burns

**Fig. 2:**
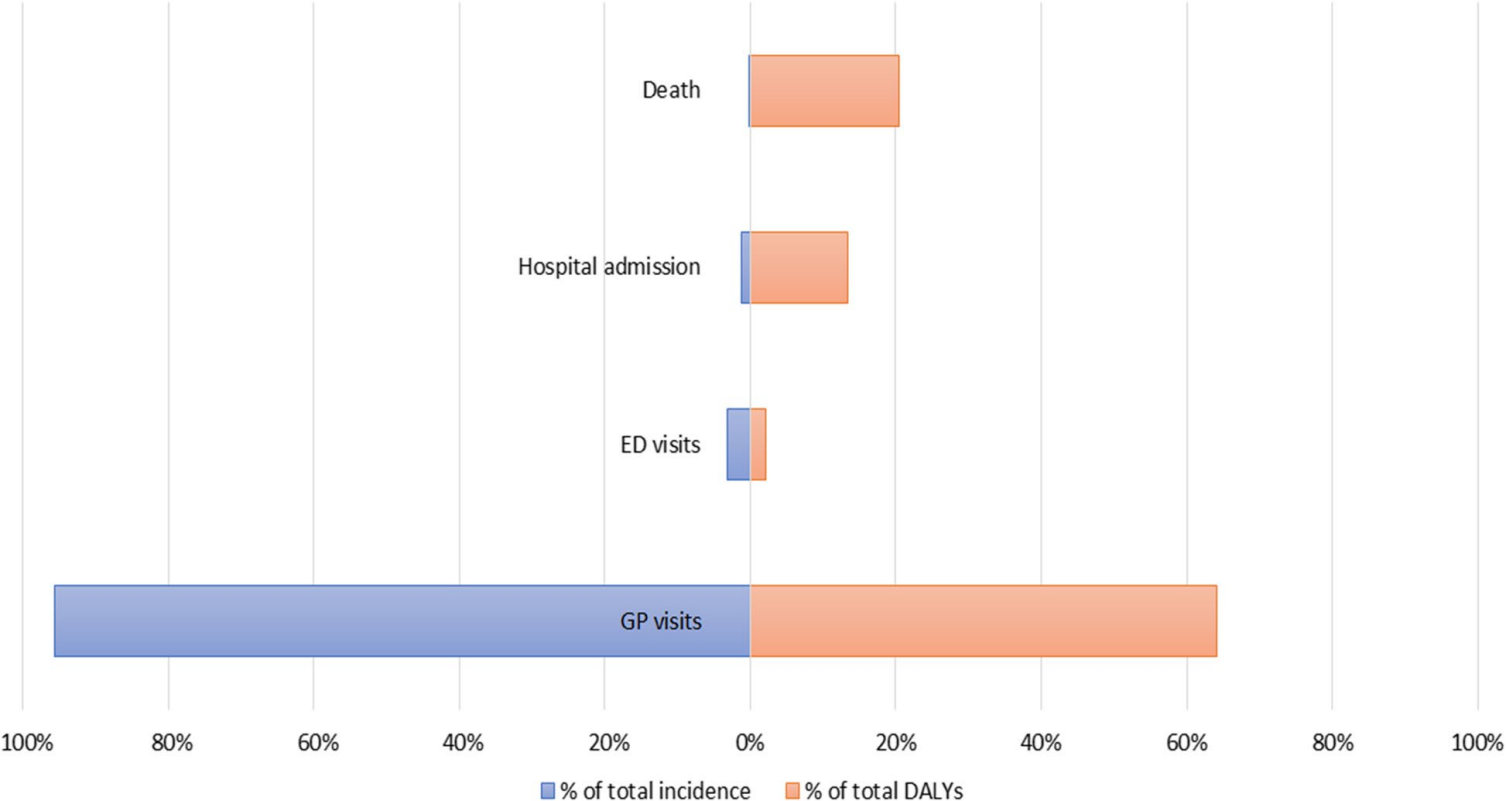
Percentage of incidence and disability adjusted life-years (DALYs) for burn injuries by level of the injury pyramid in the Netherlands in 2018. Note. ED = emergency department; GP = general practice

The data sources revealed that 113,003 persons sought medical care for burn injuries in the Netherlands in 2018. This was about 0.7% of the total Dutch population in 2018. More than half of the patients were females (58.4%). The distribution by level of the injury surveillance pyramid is shown in Fig. 2. By far the most people (*n* = 108,027; 95.6%) consulted the GP for their burn injury. About 3 % of the people with burns (*n* = 3533) were treated at the ED, and 1365 persons (1.2%) were admitted to a hospital due to their burn injury. In 2018, 78 persons died due to burns in the Netherlands.

### Burden of disease of burn injuries

The total burden of disease of burns was 9278 DALYs (0.54 DALYs per 1000 inhabitants) in the Netherlands in 2018. Non-fatal burn injuries contribute 79.6% (7385 YLDs). The average number of DALYs was 0.08 per patient, ranging from 0.06 DALYs per patient needing general practice or ED care to 24.26 DALYs per patient that died. The distribution of DALYs by levels of the injury surveillance pyramid is shown in Fig. 2. GP visits contributed most DALYs (64.0%; 5941 DALYs), followed by mortality (20.4%; 1893 DALYs), hospital admissions (13.5%; 1250 DALYs) and ED visits (2.1%; 194 DALYs).

#### Burden of disease of burn injuries according to sex

**Fig. 3:**
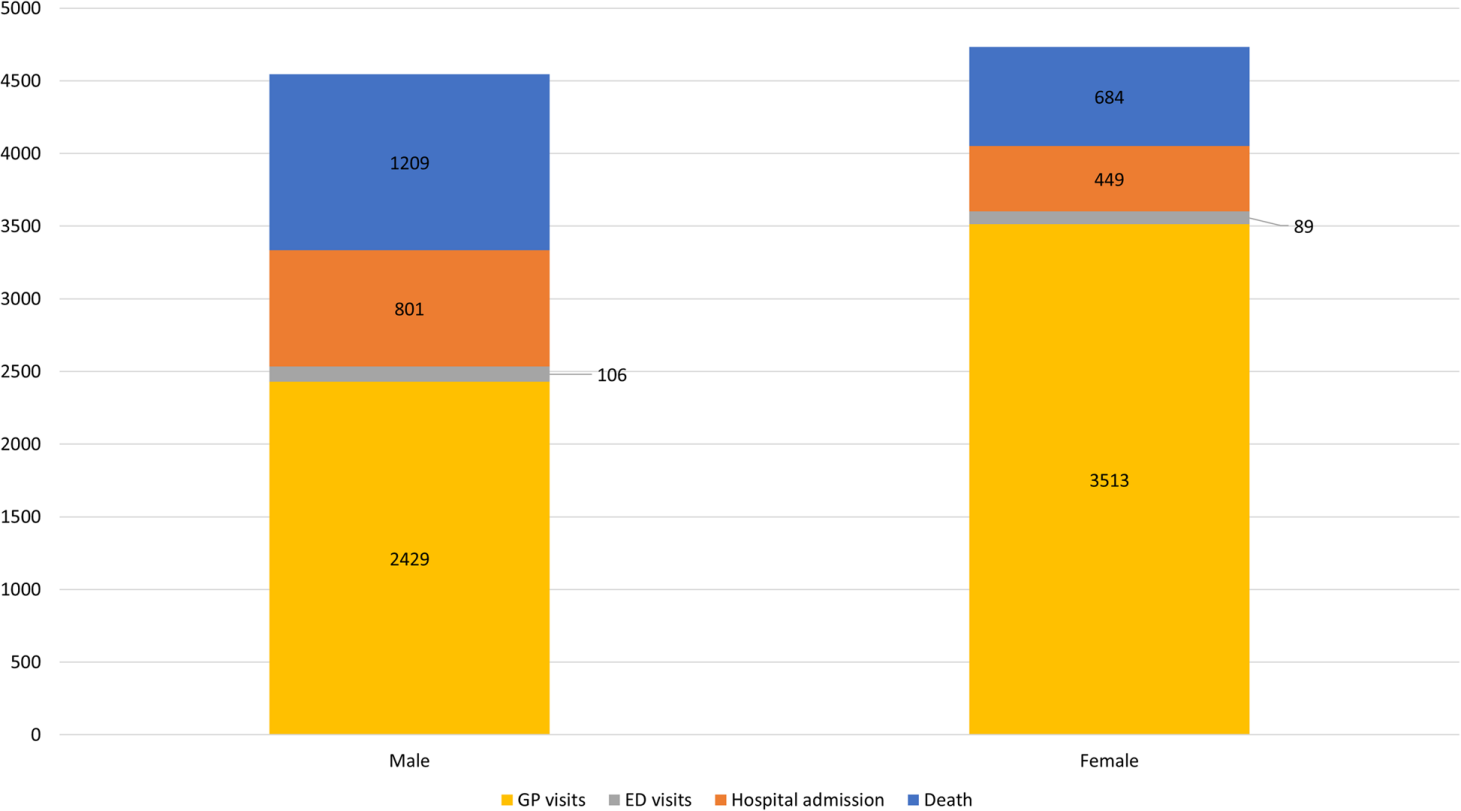
Sex specific disability adjusted life-years (DALYs) by level of the injury pyramid in the Netherlands in 2018

Overall, females were responsible for a slightly higher number of total DALYs (4734 (51.0%); versus males 4544 (49.0%)) in 2018 (Fig. 3). With regards to GP visits, the number of DALYs for females was 1.4 times higher than for males. However, the proportion of DALYs was higher for males compared to females for all other levels of the injury pyramid. The number of DALYs for males was 1.8 times higher for hospital admissions and deaths, and 1.2 times higher for ED visits. At patient level, males had a higher number of DALYs per case compared to females: 0.10 DALYs/case for males vs. 0.07 DALYs/case for females in 2018.

#### Burden of disease of burn injuries according to age

**Fig. 4:**
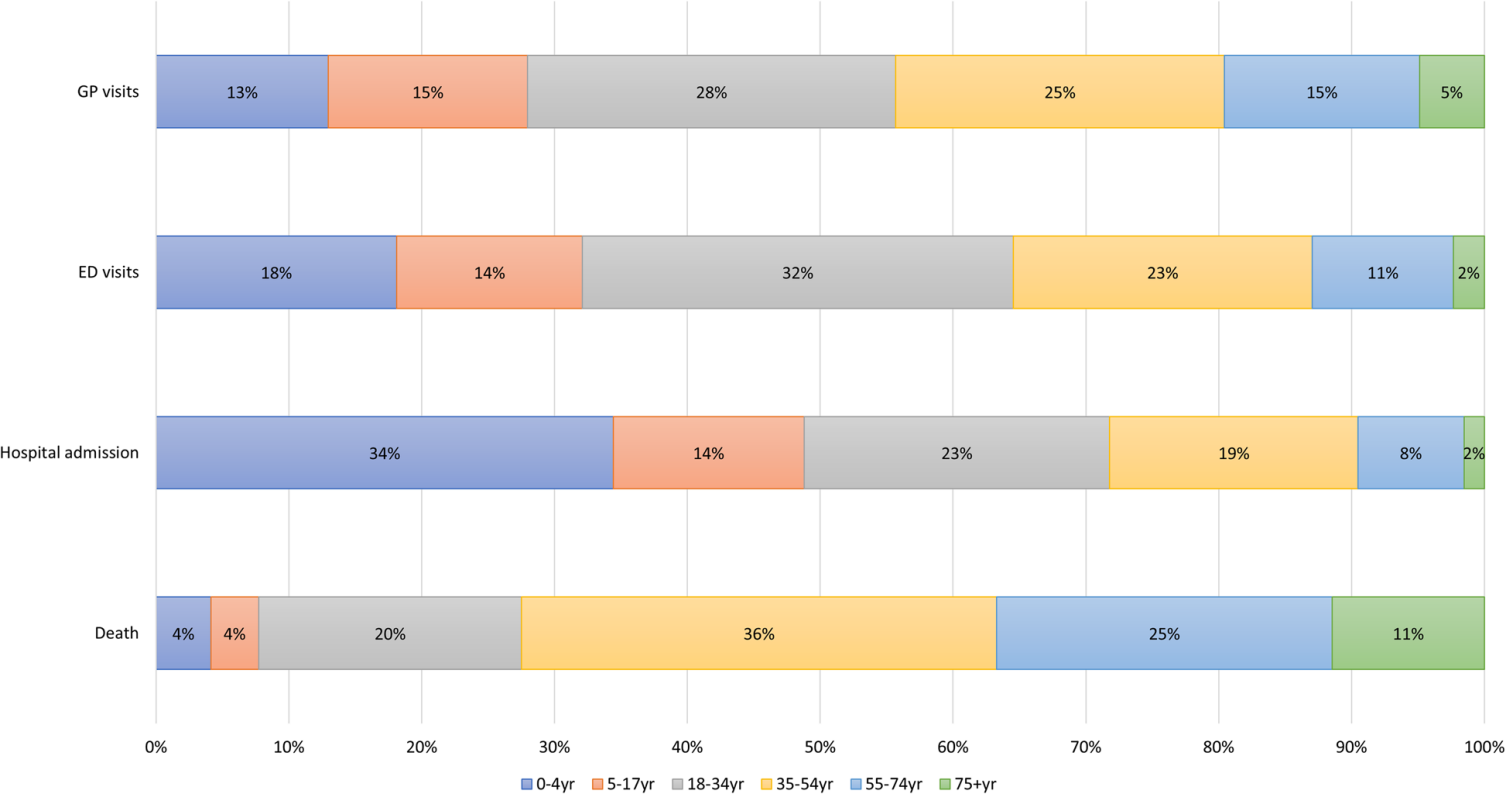
Age distribution of disability adjusted life-years (DALYs) per level of the injury pyramid in the Netherlands in 2018

The age group 35-54 yr contributed most to the total burden of disease; 2425 DALYs (26.1%), followed by the age group 18-34 yr (2371 DALYs; 25.6%) in 2018. Within children, the age group 0-4 yr old contributed more to the total DALYs compared to the group 5-17 yr old; 1313 DALYs (14.2%) and 1167 DALYs (12.6%) respectively. The age distribution of DALYs varied between the levels of the injury pyramid (Fig. 4). The highest number of DALYs at GP level (28%) and ED level (32%) came from those 18-34 years old. Young children (0-4 yr old) contributed most DALYs at hospital admission level (34%). And those 35-54 years old contributed most DALYs by death.

#### Burden of disease of burn injuries according to age and sex

**Fig. 5:**
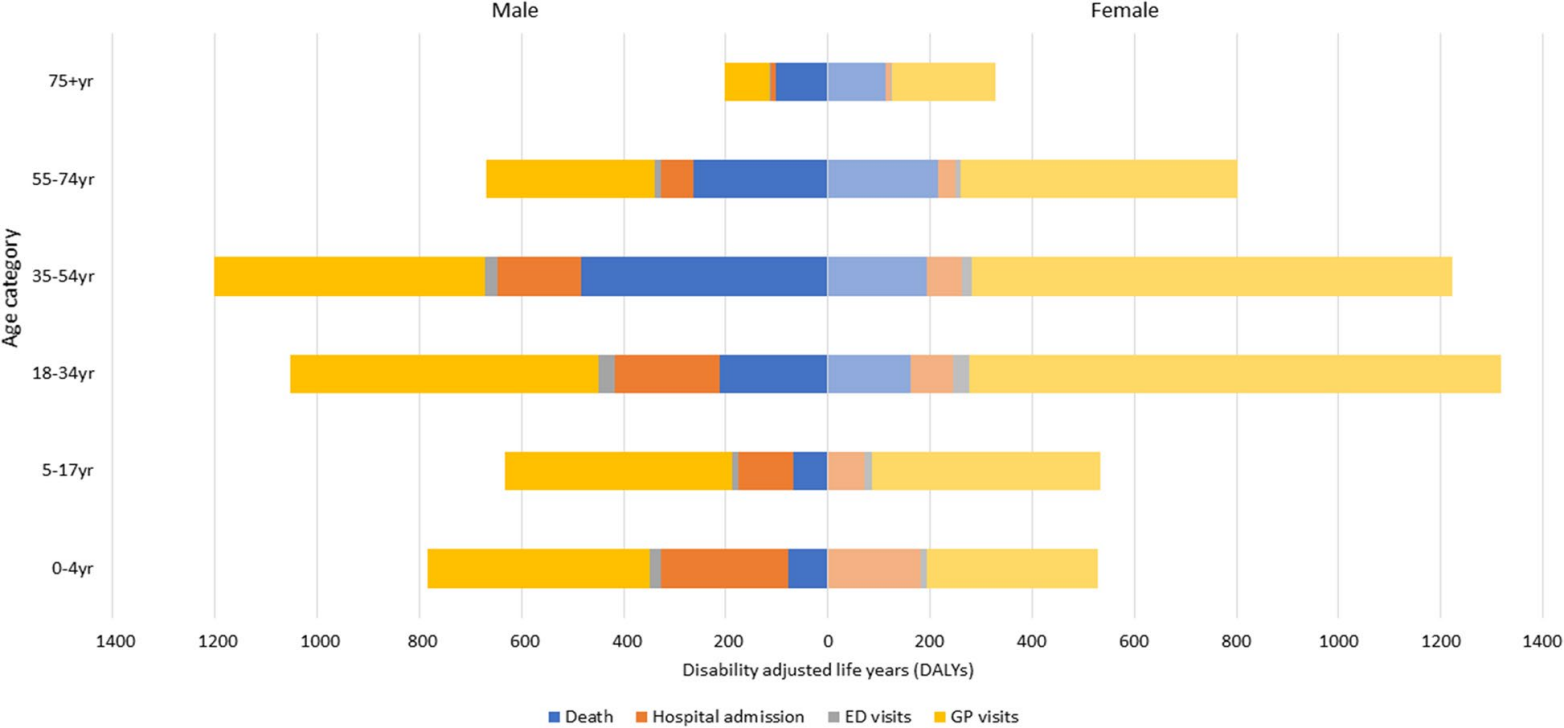
Age-and sex specific disability adjusted life-years (DALYs) by level of the injury pyramid in the Netherlands in 2018

Females 18-34 years old had the greatest total number of DALYs, namely 1319 DALYs (14.2%), whereas males aged ≥75 years old had the lowest number of DALYs: 203 (2.2%) (Fig. 5). Within children, boys aged 0-4 years were responsible for most DALYs (784; 8.5%). Overall, more DALYs were contributed by boys than girls for both age groups of children and all layers of the injury pyramid, whereas in adults, females contributed more DALYs than males in all four adult age groups. However, the number of DALYs per case were higher for males compared to females in all age groups. DALYs per case ranged between 0.08 and 0.12 in males, and between 0.06 and 0.09 in females.

### Burden of disease of burn injuries in 2014-2018

**Fig. 6:**
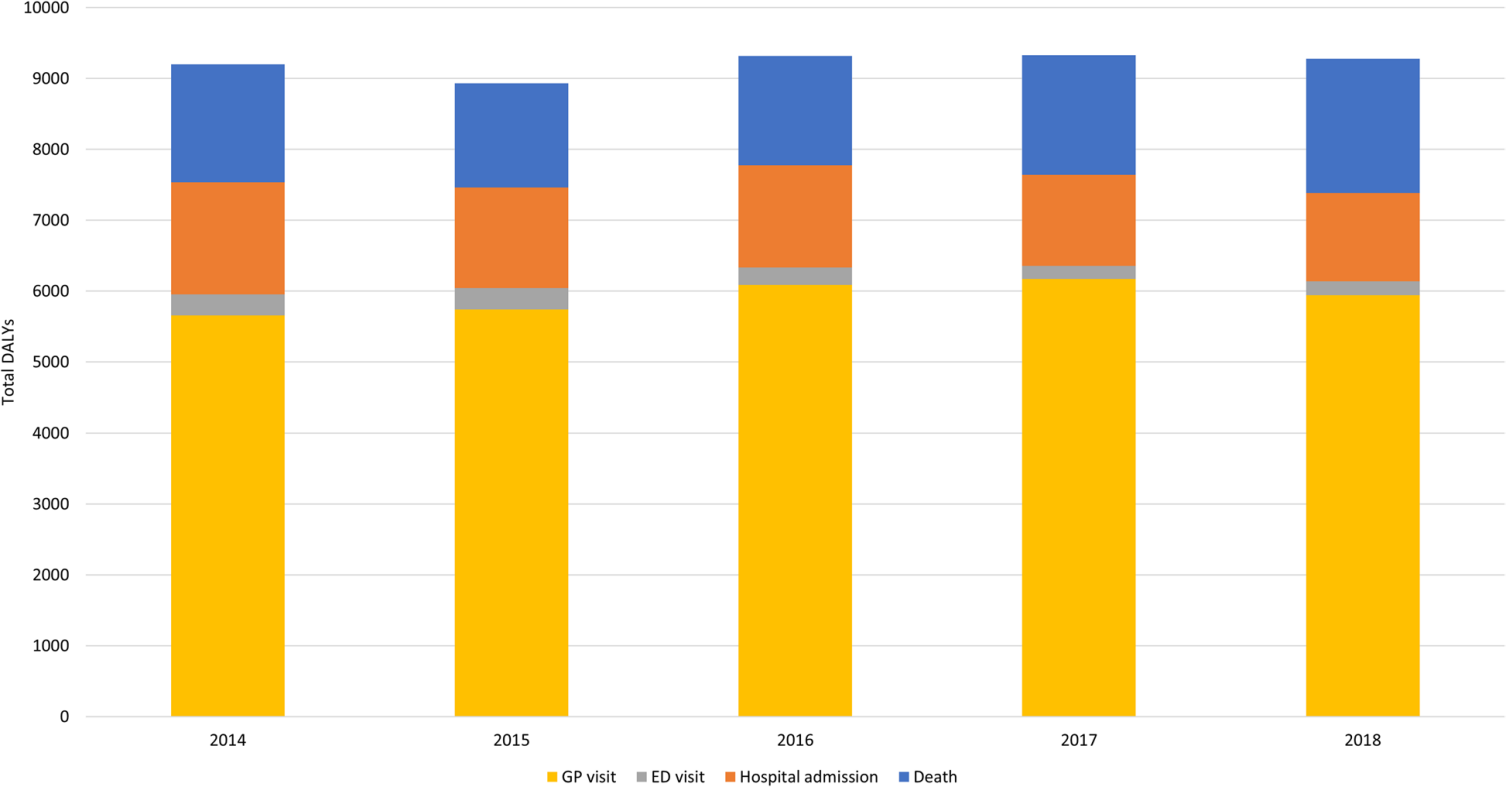
Total disability adjusted life-years (DALYs) by level of the injury pyramid in the Netherlands in 2014-2018

Figure 6 shows the DALYs by level of the injury pyramid over a 5 year period, from 2014 to 2018. Total DALYs were lowest in 2015 (DALYs: 8934) and highest in 2017 (DALYs: 9329). The difference between 2014 and 2018 was a marginal increase of only 0.8%. DALYs per 1000 inhabitants ranged between 0.53 (2015) and 0.55 (2014, 2016, 2017). The contribution of general practice was highest and stable over the years, ranging between 61.5% (2014) and 66.2% (2017). Deaths contributed 16.4% (2015) to 20.4% (2018) of the total DALYs, and hospital admissions 13.5% (2018) to 17.2% (2014).

## Discussion

When taking the full spectrum of care into account, burn injuries resulted in a total of 9278 DALYs in 2018 in the Netherlands. GP visits contributed most to the total number of DALYs, followed by mortality. The total number of DALYs was more or less similar for both sexes; it did however vary across level of the injury pyramid, with a higher proportion females at the GP level and a larger proportion of males at the other three levels. The total number of DALYs was highest for females 18-34 years old. The number of DALYs per patient were higher for males than females in all age groups. Between 2014 and 2018, the total DALYs due to burns was relatively stable.

An earlier study, based on Dutch burn centre admission data, reported a total of 771 YLDs in 2017 [18], which is almost 10 times lower compared to our findings based on the whole spectrum of care. The present study in particular shows the high contribution of DALYs from GP visits. This is partly induced by the Dutch health care system, where, just as in the United Kingdom, the GP has a gatekeeper role for secondary care. Incorporating the whole spectrum of care is thus very important to assess the overall burden of disease of burn injuries, on which decisions about priority setting and resource allocation for healthcare and prevention can be based.

Comparison of our findings to those of the GBD study are complicated as injuries are categorized following their cause of injury (e.g. fire) and by their nature of injury (e.g. burns) by the GBD. For both cause of injury as for nature of injury, YLD estimates are made, while only for the cause of injury YLL estimates are made [15]. The reason for this is that the vital registry data that are used for the GBD injury YLL estimates include cause of injury ICD codes only. The GBD study calculated a total number of 13,366 YLDs (95% CI: 8054-20,703 YLDs) for burns (nature of injury) in 2017 in the Netherlands. The uncertainty interval of the GBD estimate is quite large and our YLD estimate of 7639 in 2017 is lower. Main factors for these large differences in YLDs are the different methods used to calculate DALYs and the incidence data sources that were used as input for the YLD calculations. Differences between the two methods include the categorization of burn patients, values of disability weights, proportion of patients with lifelong disability, and recovery timeframes used [18, 19]. Besides, the GBD study used 2013 data from the Netherlands and extrapolated that to 2017, whereas in present study we used the actual 2017 data, which resulted in better substantiated burden of disease estimates.

Also, the change in burden of disease over time was different. GBD presents a slight increase [15], whereas we found a relatively stable pattern. Surprisingly other estimates were seen when comparing mortality rates. The GBD study reported 2.4 times higher YLLs than our estimates based on data from the Cause-of-Death Statistics Netherlands [44] even though a broader selection of death causes was included in our study. The GBD study uses the Cause of Death Ensemble modelling software that estimates mortality by age, sex, region, and year for specific diseases, including burn injury [45]. The premise of these estimates is that each death, also ill-defined causes of, is attributed to one single underlying cause that led to the death [46]. The redistribution of ill-defined death to burn injury related death might have resulted in a higher number of YLLs reported by the GBD study.

The present study revealed that females had a higher number of DALYs at general practice level, whereas males had a higher proportion of DALYs in each of the other layers of the injury surveillance pyramid. Thus, in general, incidence of burns is higher in females, but the burns that they sustain are less severe compared to males. This is also depicted by the higher number of DALYs per case for males compared to females. For all levels of the care spectrum taken together,, the burden of disease of burns was almost equal in both sexes. The finding that males are overrepresented at secondary care is in line with a recent systematic review on burn epidemiology that showed that males outnumbered females in incidence rates in secondary care [47]. These differences between males and females might be caused by different working environments, household chores (in particular cooking), and/or leisure time activities [47, 48] and need to be considered when burn prevention campaigns are designed. Future studies are needed to investigate the type of burns and characteristics of patients, which may further inform and improve targeted prevention among this group.

It is also notable that boys contributed more to the total burden of disease than girls. Unlike in adults, in children boys have both a higher incidence rate and sustain more severe burns. These findings are in line with an earlier epidemiological study that reported a higher rate of boys than girls admitted to a Dutch burn centre [49]. Similar findings were presented in a recent systematic review that described that the boys girls ratio was 1.56:1 [47]. Our study found that the age group 35-54 yr contributed most to the burn DALYs. Earlier studies on incidence rates described an overrepresentation (20-25%) of very young children (aged 0-4 years) in burn care [31, 50]. These studies are based on hospital admission and present study confirms this overrepresentation of young children at hospital admission level (33%). An earlier study showed that children with a young age are faster admitted for burns compared to older children and adults [49]. Children and adults are anatomically and physiologically different, which makes children more prone for more severe consequences. In comparison to adults, guidelines recommend to admit children with relatively small burns. For example, the Dutch guideline recommends to admit children with a burn size of 5% or higher, whereas for adults the burn size for referral is 10% or higher. However, when looking at the whole spectrum of care, young children contribute a smaller part of the total DALYs; about 14%. This indicates that prevention measures are very important for people at all ages.

In relation to the total burden of disease of injuries, burn injuries only account for a small part. An earlier study showed that the total burden of injury in the Netherlands was 229,322 DALYs per year in 2003-2007 [16]. Disparity in DALYs per case in burns echoes the same disparity in DALYs per case in general injuries. Interestingly, the burden of injury in general by level of the injury pyramid showed a very different pattern, with ED visits contributing much more to the total DALY compared to burns (34% vs 2%). This large difference is possibly caused by the pathology, acuteness, and type of care needed for injuries. For example, all types of bone fractures are treated in a hospital, and for most road injuries, patients are immediately transferred to an ED, whereas for burns, the injury is often less acute and can be treated by the GP. In the Dutch health care system, where the GP serves as a gatekeeper for secondary care, all care that can be provided by the GP will be provided in general practice. Besides, in the Netherlands, GP care is fully covered by the obligatory health care insurance, whereas for secondary care an annual deductible (or “own risk”; set at 385 euros in 2019 and 2020) has to be paid. For this reason, patients with mild injuries are often more inclined to visit the out-of-hours GP instead of the ED department. This was also demonstrated by a recent study that showed a decrease in minor injury related visits to emergency departments and increase in higher number of minor injury related general practice visits [51].

This study included some strengths; we included the full spectrum of care to generate an overview of the total burden of disease of burn injuries, and examined the burden of disease of different age/sex groups. Besides, present study used an improved methodology for the estimation of the burden of burn injuries. This study also included some limitations. Data were only available at an aggregated level, thereby, we could not directly correct for double counting at patient level, but we did on age/sex group level. This was the level of detail that was used for calculations in this study. However, for correction of patients treated at both the GP and in a general hospital, we only had general data at population level (0.7% of burn patients treated in general practice is generally referred to a general hospital; not a burn centre); we did not have age/sex specific subgroup data. Therefore, we lowered all sex/age specific subgroup data at the GP level by 0.7%. Another limitation includes that GP care and ED care data were extrapolated to the whole Dutch population which might have led to slight under- or overestimation of the burden of disease. However, both samples are representative for the Dutch population with respect to age and sex, and the ED care method have been validated [36, 37] and the GP care method is regularly used [24–27] and was used before for burn injuries [24]. Also, we used the same disability weights for ED visits and GP visits, as no disability weights for GP visits for burns exist. This likely has led to an overestimation of the burden of disease, as somewhat more severe burns might be treated at the ED. Another limitation was that the hospital admission data we received did not include burn size, which was needed to estimate the burden of disease. For two codes (T31 and T32) subcoding includes burn size, however, we did not receive data with this level of detail. The distribution of burn size was therefore for the whole selection (T20-32) adopted from the burn centres. However, as we are unaware of the actual severity, this might have led to a slight under- or overestimation of the burden of disease of burn injuries. Lastly, the method used is based on %TBSA burned and does not include burn depth as a factor, which is another indicator of the severity of burn injuries and its impact and recovery [17, 52]. However, as burn depth is very difficult to assess, especially by healthcare providers who do not treat burn patients on a regularly basis, often not (adequately) registered, and because %TBSA and burn depth are often correlated, we have not included burn depth in our analyses.

## Conclusion

This study provides a comprehensive overview of the burden of disease of fatal and non-fatal burn injuries in the Netherlands for the full spectrum of care in 2018. Burn injuries cause a substantial burden of disease, which was constant over the past 5 years, and to which patients who visited the GP contributed most. In addition, it was shown that among children boys 0-4 years had the highest burden of disease, and among adults, females 18-54 years old, and males 35-54 years old had the highest burden of disease due to burns. This is important information for the development of tailored burn prevention strategies, and the updated figures are recommended to be used for priority setting and resource allocation.

## Supplementary Information

**Additional file 1.** Disability weights and proportion of patients with lifelong consequences applied for the calculation of years lived with disability (YLD).

## Data Availability

The data from the different data sources can be requested from the specific institution (Nivel-PCD: https://www.nivel.nl/nl/nivel-zorgregistraties-eerste-lijn/gegevens-aanvragen-voor-onderzoek, DISS and HDR: https://www.veiligheid.nl/organisatie/monitoring-onderzoek/cijfers-nadere-analyses, DBR-R3: baarm@maasstadziekenhuis.nl). Cause-of-Death Statistics data is available at: https://opendata.cbs.nl/statline/portal.html.

## Abbreviations

%TBSA: Percentage total body surface area
DALY: disability adjusted life year
DBR-R3: Dutch Burn Repository
DISS: Dutch Injury Surveillance System
ED: Emergency department
GBD: Global Burden of Disease
GP: General practitioner
HDR: Dutch Hospital Discharge Registry
YLD: Years lived with disability
YLL: Years of life lost
