# Supplementary material for: The burden of disease of fatal and non-fatal burn injuries for the full spectrum of care in the Netherlands
Source: Arch Public Health. 2023 Jan 9;81:3. doi: 10.1186/s13690-022-01020-z (PMC9827636; doi:10.1186/s13690-022-01020-z)
Supplement: Supplementary file 1 — Additional file 1. Disability weights and proportion of patients with lifelong consequences applied for the calculation of years lived with disability (YLD). [file 13690_2022_1020_MOESM1_ESM.docx]

**Additional file 1. Disability weights and proportion of patients with lifelong consequences applied for the calculation of years lived with disability (YLD)**

| **Level of injury pyramid** | **Subgroup** | **Timeperiod** | **Disability weight applied** | **Proportion with lifelong consequences** |
| --- | --- | --- | --- | --- |
| Hospital admission^1^ | <5% TBSA burned or %TBSA unknown | 0-1 month  >1-6 months  >6-12 months  >12-24 months  >24 months | 0.173 0.098 0.082 0.102  0.046 | 20% |
|  | 5-20% TBSA burned | 0-1 month  >1-6 months  >6-12 months  >12-24 months  >24 months | 0.264 0.139 0.118 0.108  0.099 | 25% |
|  | >20% TBSA burned | 0-1 month  >1-6 months  >6-12 months  >12-24 months  >24 months | 0.497 0.262 0.231 0.163  0.122 | 39% |
| ED visits^2^ | <20%TBSA | 12 months | 0.055 | 0% |
| GP visits^2^ | <20%TBSA | 12 months | 0.055 | 0% |

^1^Based on INTEGRIS-burns method^1^.
^2^Based on INTEGRIS method^2^.

**References**

1. Spronk I, Edgar DW, van Baar ME, et al. Improved and standardized method for assessing years lived with disability after burns and its application to estimate the non-fatal burden of disease of burn injuries in Australia, New Zealand and the Netherlands. BMC Public Health. 2020;20(1):121.

2. Haagsma JA, Polinder S, Lyons RA, et al. Improved and standardized method for assessing years lived with disability after injury. Bull World Health Organ. 2012;90(7):513-521.
